# Impact of the Childcare Physical Activity (PLAY) Policy on Young Children’s Physical Activity and Sedentary Time: A Pilot Clustered Randomized Controlled Trial

**DOI:** 10.3390/ijerph18147468

**Published:** 2021-07-13

**Authors:** Monika Szpunar, Molly Driediger, Andrew M. Johnson, Leigh M. Vanderloo, Shauna M. Burke, Jennifer D. Irwin, Jacob Shelley, Brian W. Timmons, Patricia Tucker

**Affiliations:** 1 Health and Rehabilitation Sciences Program, Faculty of Health Sciences, University of Western Ontario, London, ON N6A 3K7, Canada; mszpuna@uwo.ca (M.S.); ajohnson@uwo.ca (A.M.J.); lvanderloo@participaction.com (L.M.V.); sburke9@uwo.ca (S.M.B.); jenirwin@uwo.ca (J.D.I.); jshelle6@uwo.ca (J.S.); 2 School of Kinesiology, Faculty of Health Sciences, University of Western Ontario, London, ON N6A 3K7, Canada; mdriedig@uwo.ca; 3 School of Health Studies, Faculty of Health Sciences, University of Western Ontario, London, ON N6A 3K7, Canada; 4 Child Health & Evaluative Sciences, The Hospital for Sick Children, Toronto, ON M5G 1X8, Canada; 5 Faculty of Law, University of Western Ontario, London, ON N6A 3K7, Canada; 6 Child Health & Exercise Medicine Program, Department of Pediatrics, McMaster University, Hamilton, ON L8S 4L8, Canada; timmonbw@mcmaster.ca; 7 School of Occupational Therapy, Faculty of Health Sciences, University of Western Ontario, London, ON N6A 3K7, Canada

**Keywords:** policy, childcare, physical activity, sedentary time, young children, accelerometry, intervention, early childhood educators

## Abstract

*Background*: The importance of daily physical activity is crucial for healthy development during the early years. Currently, a formal written physical activity policy is lacking in Canadian childcare centers, but holds promise for offering consistent physical activity opportunities. With eight recommendations, the Childcare PLAY policy is an evidence-informed, institutional-level document, targeting children’s physical activity, outdoor play, and sedentary time. The purpose of this study was to examine the impact of the Childcare Physical Activity (PLAY) policy on the physical activity and sedentary time of young children (18 months–4 years) in childcare. *Methods*: Nine childcare centers in London, Ontario participated in the cluster, randomized controlled trial. The centers in the control condition (*n* = 4) continued their typical daily routines, while the centers in the intervention condition (*n* = 5) implemented the PLAY policy for eight weeks. To assess physical activity levels, toddlers and preschoolers wore ActiGraph wGT3X-BT accelerometers for five consecutive days during childcare hours, at baseline, mid- and post-intervention, and at the six-month follow-up. Raw accelerometry data were converted to 15 s epochs, and age- and device-specific cut-points were applied. The participants with two or more days of at least 5 h/day of wear-time at baseline, and at one additional time point, were included in the linear mixed-effects models. An adjusted alpha (*p* < 0.017) was used to account for multiple comparison bias. *Results*: A total of 148 children (31.92 ± 7.41 months) had valid accelerometry data. The intervention resulted in a significant increase in light physical activity among the participants in the experimental group at the six-month follow-up (+1.07 min/h, an 11.16% increase; *p* = 0.0017). The intervention did not have a statistically significant effect on the total physical activity, moderate-to-vigorous physical activity, or sedentary time. *Conclusions*: The findings indicate that the Childcare PLAY policy was effective at increasing the toddlers’ and preschoolers’ light physical activity. This pilot intervention appears promising for supporting some improved movement behaviors among children in childcare settings; however, additional investigations are needed to explore the feasibility and effectiveness with larger and more-diverse samples.

## 1. Background

Physical activity participation in early childhood (<5 years) is necessary for healthy growth and development [1,2]; yet, research shows insufficient activity levels [3] and rising rates of sedentary time among this cohort [4]. Regular physical activity supports the maintenance of a healthy body weight [1], and is associated with improved cardiometabolic and psychosocial health [2]. In contrast, the time spent sedentary is associated with adverse outcomes, including increased adiposity and decreased overall health [5]. According to Reilly et al., supporting healthy movement behaviors among young children increases the chance that they will engage in regular physical activity as adults [6]. Therefore, interventions targeting toddlers’ (1–2 years) and preschoolers’ (3–4 years) physical activity may be an effective strategy for promoting positive health trajectories into later adolescence and adulthood. 

The Canadian 24-hour movement guidelines for the early years [7,8] offer guidance on the appropriate daily movement behaviors for young children. These recommendations are particularly important, and should be considered by those who care for young children (e.g., parents/guardians, early childcare educators (ECEs)). The guidelines state that children aged 1–4 years should participate in at least 180 min of physical activity per day, and for those over two years of age, at least 60 min of this should constitute moderate-to-vigorous-intensity physical activity (MVPA), or energetic/active play. Prolonged sitting should be limited to no more than 60 min at a time, regardless of age. Screen-viewing is not recommended for children under two and should be limited to 60 min/day for children 2–4 years of age [7,8]. Other countries (i.e., Australia, United Kingdom) and the World Health Organization [9] have also adopted similar guidelines, suggesting a demand exists for age-specific physical activity targets. Unfortunately, research suggests that many children may not be sufficiently active [3] to meet these guidelines, and these trends are particularly observed among children who are enrolled in center-based childcare [10,11]. A recent systematic review found that toddlers’ and preschoolers’ (*n* = 13,956) levels of MVPA ranged from 1.3 to 22.7 min/h during childcare hours [12], revealing that there is great variability in physical activity engagement, and this is likely a consequence of the device employed and the cut-points applied to identify activity intensities. However, what is clear is that many children are not engaging in adequate physical activity and too much sedentary time (as high as 41 min/h) [13] in childcare settings, and these findings are not in accordance with the early years recommendations. 

Children often participate in developmentally appropriate sedentary behaviors in childcare settings (e.g., reading, coloring, circle time), but engagement in screen-viewing has also become more prevalent [14]. Excessive screen-viewing is problematic among young children, as it has been noted to lead to irregular sleep patterns [15], and increase the risk of obesity and depression [16]. In line with the Canadian recommendations, interventions that are aimed at promoting physical activity and reducing screen-viewing should be tailored for center-based childcare settings, as well as for ECEs, who are very influential in shaping young children’s behaviors [17]. ECEs are responsible for daily programming [18], which includes incorporating physical activity affordances into the daily scheduling for young children. Fostering strong physical activity-related knowledge among ECEs is fundamental, to ensure young children are engaging in healthy movement behaviors during their time spent in care. 

In Canada, provinces/territories are responsible for regulating childcare; as a result, substantial variations in movement behavior direction exist. More specifically, only four Canadian provinces (Nova Scotia, British Columbia, Northwest Territories, and Nunavut) currently mention physical activity in their regulations [19,20], and British Columbia is the only province that regulates screen-time [20]. With the little (and varied) guidance for programming physical activity opportunities across the country, the responsibility falls primarily to childcare centers and ECEs to implement physical activity opportunities into their already demanding curriculums. Research exploring the prevalence of institutional-level policy at childcare centers (*n* = 1158) in Canada found that very few have a policy that references physical activity (~44%) or screen-viewing (~29%) [21]. The implementation of movement-related policy is alarmingly low, as studies have acknowledged the potential effectiveness of a physical activity policy in childcare centers [22,23,24], based on the success in other countries (e.g., United States [24,25,26] and China [27]). For example, researchers in China found that children receiving a physical activity policy intervention achieved nearly 60 min/day of MVPA, while children not receiving the policy only achieved 31 min/day of MVPA [27]. Similarly, Dowda and colleagues (2009) examined the effects of various policies (e.g., daily teacher-facilitated physical activity) and characteristics (e.g., outdoor playground size, available playground equipment) within preschools (*n* = 20), and noted that children attending *physical activity promoting* preschools in the United States (e.g., centers with higher-quality physical activity standards, characteristics, and/or policies) accumulated more time in MVPA per day, and less time engaged in sedentary behaviors, compared to non-physical activity-promoting preschools [28]. Considering the available evidence in the literature, researchers in the field have emphasized that childcare policies should focus on incorporating increased amounts of outdoor play [29], structured (e.g., teacher-led) physical activity [30], and limiting screen-viewing unless it is required for educational purposes [31]. As no consistent regulations currently exist in Canada, researchers are challenged with the task of piloting interventions that are aimed at increasing physical activity and limiting screen time. This is important, as the implementation of policy in childcare has the potential to equalize physical activity affordances, so that all young children in care receive the same opportunities to be active.

The purpose of the Childcare PLAY policy pilot study was to examine the effectiveness of an evidence-based, stakeholder-informed, written physical activity and sedentary time policy on young children’s movement behaviors. Policy, in the childcare context, represents a strategy to equalize affordances in physical activity opportunities; policy *levels the playing field*, to ensure young children are receiving uniform opportunities. It was hypothesized that the children allocated to the experimental condition would exhibit increased total physical activity (TPA) compared to the children whose centers did not implement the policy. The secondary objectives included determining whether the policy increased young children’s light physical activity (LPA), MVPA, and decreased their sedentary time.

## 2. Methods

### 2.1. Description of the Childcare PLAY Policy

The policy encompassed eight statements targeting physical activity, outdoor play, and sedentary/screen time. Example policy items included statements that encouraged children’s daily engagement in higher-intensity energetic play with a goal of accumulating a minimum of 40 min throughout the day; a variety of indoor and outdoor physical activities; and both child-directed (i.e., unstructured) and teacher-facilitated (i.e., structured) active play. In line with the Canadian 24-hour movement guidelines for the early years (with time recommendations adjusted to account for the typical childcare duration), the policy was created in collaboration with the London, Ontario childcare community, physical activity researchers, and policy experts. The goal of the proposed policy was to complement Ontario’s current regulations (i.e., two 1-h outdoor playtime sessions during childcare hours [32]), and offer ECEs feasible direction in regard to offering young children increased physical activity affordances. See Supplementary Materials Figure S1 for the Childcare PLAY policy. 

### 2.2. Study Design and Recruitment

A pilot, single-blind, cluster-randomized controlled trial (RCT) with four data collection time points (i.e., baseline, mid-, post-intervention, 6-month follow-up) was conducted. The director of randomly (*n* = 9) selected childcare centers was contacted via telephone and once all nine centers agreed to participate and provided consent, centers were assigned to the control or experimental condition via a block randomization. All recruitment and randomization procedures took place between July and August 2018 with baseline data collection staggered by center in September and October 2019. Childcare centers assigned to the experimental condition were required to implement the Childcare PLAY policy, while control centers continued their typical daily regimes. The research assistants responsible for conducting assessments and data collection were unaware of group assignments, thus ensuring a single-blind design. A detailed methodological account has been published elsewhere [33]. The study and related documents received approval from the Health Sciences Research ethics board at the University of Western Ontario (REB #111890) and was registered in the Clinical Trials Registry provided by the US National Library of Medicine (registration number: NCT03695523; registration date: 04 October 2018; https://clinicaltrials.gov/ (accessed on 9 July 2021).

### 2.3. Participants

**Figure 1:**
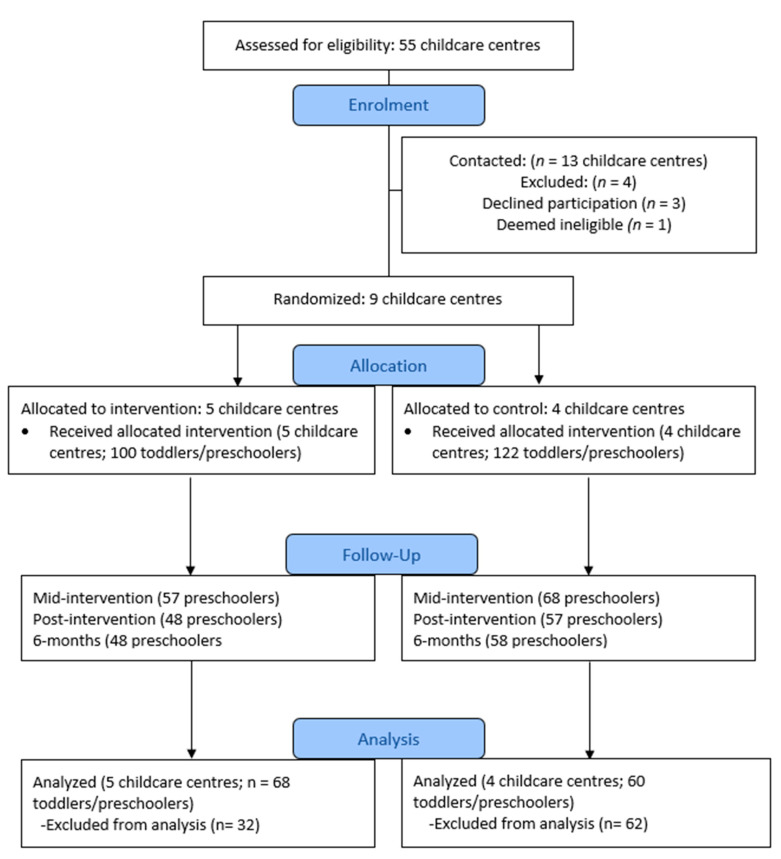
CONSORT flow diagram for the PLAY policy intervention. *Note.* No centers were lost to follow up or dropped out of the intervention. Toddlers and/or preschoolers were excluded for the following reasons: inadequate wear-time, withdrawal from childcare, device malfunction, and absence from childcare center during data collection periods.

Parents/guardians of typically developing (i.e., free from chronic disease, disability and/or developmental delay) toddlers (18 months–2.4 years) and preschoolers (2.5–4 years) from consenting childcare centers in London, Ontario were invited to participate. Children were eligible to participate if they had a parent/guardian fluent in English who provided consent. A complete list of inclusion and exclusion criteria is reported elsewhere [33]. Based on the sample size calculation, the goal was to recruit 218 children from eight childcare centers [33]. Once randomization of childcare centers into control and experimental conditions occurred, there was a substantially higher number of participants in the control condition. Thus, one additional center was randomly selected, recruited, and allocated to the experimental condition to ensure an equal dispersion among groups. This additional center was selected using the same selection, recruitment, and randomization processes employed in the initial randomization. Please see Figure 1 for the study consort diagram. 

### 2.4. Data Collection

All data collection took place between September 2018 and June 2019, with the intervention being implemented in a staggered format (e.g., pre-intervention, week 0; mid-intervention, week 4; post-intervention, week 8; and at 6-month follow-up). ECEs in the experimental condition documented their adherence to each of the policy components in a daily implementation log. Prior to intervention implementation, participating ECEs from the experimental condition attended a 30-min intervention training session, which provided a detailed summary of the study design, the importance of physical activity for young children, an overview of the 8 policy components and their implementation, and study tools (e.g., questionnaires and accelerometer on/off logs). Upon completion of the study, staff members from the childcare centers in the control condition were able to request a copy of the written physical activity policy and its associated training. Research staff visited participating centers weekly to distribute accelerometers and study tools (e.g., daily accelerometer wear-time logs). 

### 2.5. Instruments and Tools

**Accelerometers.** Toddlers and preschoolers in both the control and experimental conditions were required to wear their initialized ActiGraph GT3x-BT accelerometer during childcare hours only (i.e., from the start of arrival until end of childcare day) for five consecutive days (i.e., Monday–Friday) at the four different time points throughout the study. Research assistants or trained childcare staff fastened the accelerometers to the right hip of each child using an adjustable neoprene belt upon arrival at childcare in the morning and removed the device and belt before departure at the end of the day. Staff recorded the wear-time (e.g., on/off times) of the devices for each child in a log. Accelerometer data were collected in raw form to minimize error associated with future data manipulations, and to capture children’s brief and sporadic movement patterns [34]. Accelerometer data were re-integrated into 15-s epochs prior to data analysis, in line with studies in the field (e.g., appropriate for this age group) [3,35]. Similar to previous research [36], the Pate et al. cut-points were applied to children’s movement data to decipher intensities, as follows: per 15-s epoch, counts ranging from 0 to 24 were defined as sedentary time, 25–420 counts were defined as LPA, and >420 counts were defined as MVPA [37].

**Anthropometric measurements.** Anthropometric measurements were taken at baseline (week 0) for all participating children. Trained research assistants who were blind to group assignment took all measurements, and children were asked to remove shoes and any heavy clothing (e.g., jackets) beforehand. Weight was measured to the nearest 0.1 kg using a Tanita scale (model #700-TBF300GS body fat analyzer w/goal setter), height was measured to the nearest 0.1 cm using a stadiometer (i.e., Seca 214 “road rod” portable stadiometer), and waist circumference was measured to the nearest 0.1 cm using a measuring tape. Age- and sex-specific BMI percentiles were computed using the Centers for Disease Control and Prevention (CDC) reference values [38]. Verbal assent from children was obtained before measurements were taken.

**Demographic questionnaire.** Parents/guardians of participating children were asked to complete a demographic questionnaire at baseline. This questionnaire was used to collect information such as the following: age, sex, and ethnicity of the child, family income, parent/guardian education level, and participation in any extracurricular activities by the toddler or preschooler outside of childcare hours. 

### 2.6. Data Analysis

Descriptive statistics were calculated to describe participant demographic data. To ensure age and sex comparability between groups, independent sample *t*-tests and a Pearson Chi-square test were completed.

Accelerometer data were downloaded using device specific software (ActiLife version v6.13.4; https://actigraphcorp.com/support/software/actilife/ (accessed on 12 July 2021), and only participants with a minimum of two valid days (where 300 min (i.e., 5 h) of consecutive wear-time equated a valid day) at baseline and one additional time point were retained for analysis. In line with previous research within this population [39], non-wear-time was defined as 10 min of consecutive zeros. Applying this parameter ensured children’s movement during naptime was not mistaken for movement or lack thereof (i.e., sedentary time). To explore the effect of the intervention over time on accelerometer-measured activity, a maximum likelihood linear mixed-effects model was utilized, with group (experimental versus control), time (baseline, mid- and post-intervention and 6-month follow-up), and sex entered as fixed effects. Age was entered into the models as a covariate, and childcare center was included as a random effect in all models. Linear mixed-effects modelling reduces concerns regarding missing data on the dependent variables (i.e., the analysis uses all available data without the need for interpolation). Interaction terms were evaluated using interaction plots, which allowed us to visualize the differential impact of time within each of the two groups. Further to this, all possible comparisons among the time periods were evaluated, and effects were compared between groups. All statistical analyses were performed using R version 4.0.5 ([40] with linear mixed-effects analyses conducted using the nlme [41] and car [42] packages. All possible comparisons amongst the time periods were assessed using the emmeans package [43]. A Bonferroni correction was used to adjust for multiple comparison bias; three analyses were performed (sedentary time, LPA, and MVPA), and so the comparison alpha was set to 0.05/3 = 0.017. TPA is an aggregate variable that is a linear combination of LPA and MVPA and was therefore analyzed within a separate family of comparisons to sedentary time, LPA, and MVPA (i.e., alpha was not adjusted for this comparison).

## 3. Results

### 3.1. Description of Sample

A total of 219 toddlers and preschoolers (31.92 ± 7.41 months; 88 girls, 80 boys) were enrolled in the Childcare PLAY policy study. Once the accelerometer wear-time parameters (two valid days at two+ time points) were applied, 148 participants were retained for analysis (31.93 ± 7.74 months; 64 girls, 56 boys). Of those retained, no statistically significant differences between the groups, in terms of age, sex, or wear-time, were noted. See Table 1 for full participant demographics.

### 3.2. Effects of Childcare PLAY Policy Intervention on Participants’ Activity Levels

**Figure 2:**
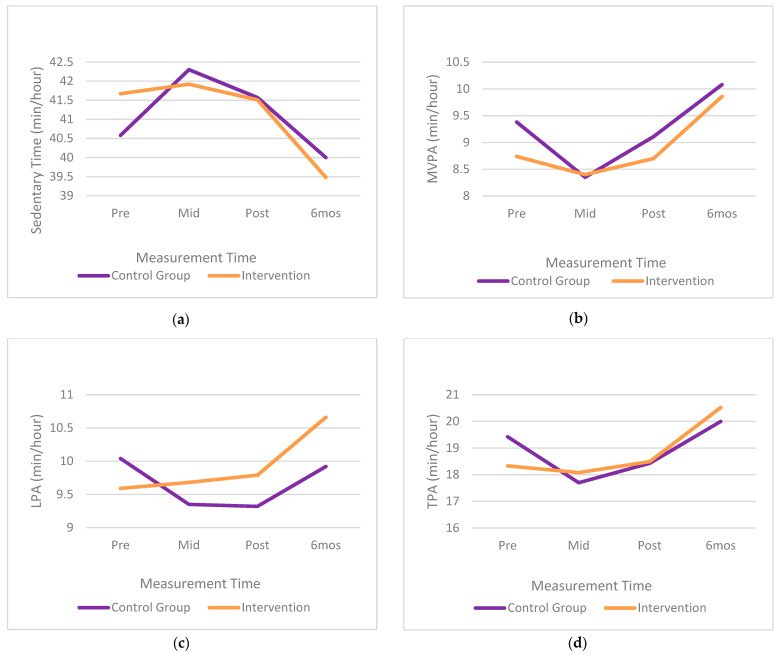
(**a**–**d**). Interaction plots for young children’s activity levels (**a**) sedentary time, (**b**) moderate-to-vigorous physical activity, (**c**) low physical activity, (**d**) total physical activity.

The means and standard deviations for physical activity intensities and sedentary time, separated by time and group, are presented in Table 2. The children in the experimental group significantly improved their LPA, by 1.07 min/h, which is an 11.16% increase, compared to those in the control group, at the six-month follow-up, χ^2^(3) = 16.68, *p* = 0.00082. No effect of the intervention (i.e., no significant interaction) was found (α = 0.017) for sedentary time, χ^2^(3) = 5.56, *p* = 0.13, MVPA, χ^2^(3) = 2.28, *p* = 0.52, or TPA, χ^2^(3) = 5.56, *p* = 0.13. The statistically significant interaction for LPA is presented in Figure 2. From this figure, it is apparent that the children in the intervention group demonstrated a steady improvement over the course of the intervention, and into the six-month follow-up. The improvement was statistically significant at the six-month follow-up, when compared with the scores at baseline (t(273) = 5.12, *p* < 0.0001), mid-intervention (t(273) = 4.50, *p* = 0.0001), and post-intervention (t(273) = 3.77, *p* = 0.0012). No post hoc comparisons were statistically significant for the children in the control group.

## 4. Discussion

The purpose of this study was to examine the impact of the Childcare PLAY policy on the device-measured physical activity and sedentary time of young children in childcare. Given that childcare centers play a significant role in shaping young children’s activity behaviors and health trajectories, it was appropriate to pilot the policy within these venues. The results of this study suggest that the policy was successful at increasing the participants’ LPA activity over the course of the intervention, and into the six-month follow-up. However, no significant results were obtained for MVPA, TPA, and sedentary time. Several findings warrant discussion.

To date, a small body of research has been conducted that explores the effects of childcare center policies on children’s movement behaviors, and policy implementation [44]; rather, the majority of studies that have transpired in this field have investigated policy uptake (e.g., examining how many childcare centers follow and/or have policy in place) [21,23,45,46]. For example, in New Zealand, Gerritsen et al. (2016) found that only 35% of participating licensed childcare centers (*n* = 237) had a physical activity policy in place, and none mentioned screen-viewing [23]. Similarly, in Australia, researchers found that only 58% of childcare services (*n* = 215) had written physical activity policies [44]. With regard to studies that include the implementation of specific policies (e.g., detailed written policies, which explicitly target behavior change among young children during childcare hours), a small handful of policy interventions have transpired that focus on physical activity [24,25,26,27,47]. Although further research is needed, the results of existing physical activity-targeted policy interventions that are similar to the Childcare PLAY policy, demonstrate the potential of imposing higher-level prescriptions (e.g., physical activity and sedentary behavior recommendations, with explicit detail and more direction for ECEs) for promoting healthy movement trends among young children. 

The Childcare PLAY policy was designed with the intention to provide ECEs with clear direction (e.g., through the written policy statements) regarding how to offer sufficient physical activity opportunities. Given the efforts that have been put forth by the research team, to create a suitable policy that would achieve ECE buy-in, the study results showed that the children in the experimental group experienced an increase in LPA (11%) at the six-month follow-up. The observed increase in the experimental group children’s LPA is consistent with LaRowe and colleagues’ (2016) policy study. LaRowe and colleagues’ policy resulted in increased time spent in LPA (*r* = 0.35, *p* < 0.05) among children. In addition, their intervention also resulted in a decrease in sedentary time after 12 months (−4.4 ± 14.2% time, −29.2 ± 2.6 min, *p* < 0.02). It is important to note that LaRowe and colleagues’ sample of young children (*n* = 456) was much larger than what was included in the present PLAY study, but they did not include a comparison group [48]. Although LPA does not offer the same health benefits as MVPA [2], children enrolled in the present study showed a sustained increase in LPA into the six-month follow-up, indicating positive improvements. 

The Childcare PLAY policy intervention placed an emphasis on outdoor time; specifically, the importance of offering children increased outdoor periods. Outdoor time was embedded as a part of the PLAY policy because studies have shown a correlation between outdoor time and physical activity among young children [49,50], and a correlation between outdoor time and MVPA [29]. It was anticipated that the policy would result in increased levels of MVPA, as the study findings from Bower et al. showed that children attending childcare centers with an existing physical activity policy, engaged in greater amounts of MVPA (15%), compared to children in childcare centers with no policy (9%) [25]; however, similar results were not apparent in the present study. Incorporating multiple outdoor periods has proven effective in previous research studies (i.e., shorter, more frequent play periods, as opposed to two 1-h periods) transpiring in childcare [51,52,53,54]. For example, Tucker and colleagues’ (2015) intervention included more frequent outdoor periods (i.e., four 30-min periods), and this intervention was associated with increased MVPA [54]. In addition, a study by Wolfenden and colleagues (2016), exploring the effects of three outdoor periods, also showed promising results; however, this study took place in Australia, which does not experience the same degree of inclement weather that occurs in Canada [53]. Relevantly, the present intervention took place during the winter months, which had a large influence on ECEs’ abilities to implement these more-frequent outdoor periods. The process evaluation of the Childcare PLAY policy revealed low adherence (e.g., 12% implementation rate of multiple outdoor periods [55]), and the interview participants revealed similar challenges. Thus, the inclement weather during the policy implementation period, and the challenge to implement as noted by ECEs [55], could account for the lack of significant changes observed in the MVPA and TPA outcomes between the groups. As such, it is important that future research explores how incorporating multiple outdoor periods can be made feasible in countries that experience weather variability (e.g., inclement conditions), due to existing evidence that multiple outdoor periods can increase higher-intensity activity.

As part of the PLAY process evaluation [55], childcare providers reported high fidelity to breaking up long periods of sedentary time, which was included as a policy item; however, no statistically significant changes were observed. Research conducted in childcare settings has shown that children are more likely to be sedentary while indoors compared to outdoors [29,56], due to the challenges with engaging children in activity while inside childcare settings. This lack of change in sedentary levels could also be linked to the inclement weather observed during the policy implementation period, since colder temperatures and snow can preclude outdoor play [29], which has been previously identified as more conducive to physical activity. In addition, ECEs have previously noted their hesitation to engage children in higher intensity play while indoors (e.g., safety concerns) [17], and these findings are consistent with the present study [55]. ECEs who participated in the present intervention, noted their fear that children would knock or bump into things while indoors, which may have promoted them to use indoor time for other tasks required in their daily curriculums (e.g., reading) that are more sedentary by nature [55]. Finally, ECEs are required to get creative while indoors, due to the many obstacles (e.g., tables, chairs, other children) that are common to the childcare environment, which may have contributed to the lack of significant results concerning sedentary time. 

### Limitations

The key strengths of the present study include the RCT methodology that was employed, the unique evidence-based and stakeholder-informed policy components, and the use of accelerometers to measure movement behaviors. Additionally, the Childcare PLAY policy was delivered by ECEs, and was designed to offer flexible implementation of the policy into their unique schedules/programming in respective childcare centers. Despite these noted strengths, this study is not without limitations. A number of participants were lost at the six-month follow-up, due to withdrawal from childcare centers or from advancing to kindergarten care (childcare is a transient environment). It seems possible that with a larger sample, paired with executing the policy over a longer period of time (e.g., over the course of a year, including all seasons), the effects of the policy may show more promising results for increasing young children’s physical activity. Moreover, accelerometer wear-time/fidelity was challenging, thus resulting in a smaller sample size than intended. It was anticipated that a greater number of children would have met the wear-time criteria (i.e., at least 300 min on two valid days). Implementing the Childcare PLAY policy with a larger, more diverse sample may offer a more accurate picture of policy effectiveness, and increase the number of preschoolers/toddlers included in analyses. Additionally, more cases of incomplete or missing data were apparent among the toddler samples, which indicates that additional measures should be taken to enhance the feasibility of assessing young children’s activity levels (e.g., device adherence, accelerometer placement). Finally, adherence rates to the study may have been a limitation. ECEs had low adherence (12%) with implementing one of the policy items (e.g., shorter, more-frequent outdoor play periods [55]). Although high adherence (93%) was reported to other policy items (i.e., limiting children’s exposure to screen-based technology), the shorter, more-frequent outdoor play periods were especially important for this intervention. Despite these limitations, the present study offers evidence of the impact of a childcare policy on children’s LPA; however, additional research, with a larger sample, is necessary to confirm these findings. 

## 5. Conclusions

The Childcare PLAY policy was found to be effective at improving young children’s LPA. While positive activity behavior trends were also apparent for both TPA and MVPA, the lack of a significant effect is likely due to the small sample size. As a large proportion of young Canadian children are enrolled in childcare, it is imperative that further policy research be conducted in order to identify how these settings can provide adequate opportunities for physical activity participation, and how policy can offer guidance to ECEs and enforceable programming for childcare centers to follow. Considering the policy was well-received by childcare centers and ECEs [55], and supported increased LPA among children, additional investigation (i.e., increasing the sample size, implementation during a different season) is needed to further explore the Childcare PLAY policy’s impact. Childcare policies hold great potential for province- and nation-wide adoption, and may be a promising strategy to prevent adverse health outcomes among young Canadians.

## Figures and Tables

**Table 1:** Descriptive characteristics of enrolled toddlers and preschoolers.

Variable	Control	Experimental	*p*-Value
Age (years), M (SD)	2.66 (0.58)	2.65 (0.63)	0.36
Sex (male, female), n	40, 45	40, 43	0.88
Body mass index percentiles, M (SD)	51.6 (30.4)	53.3 (31.1)	0.79
Hours spent in childcare/week (hours)			0.004
<10	2	2	
10–19	2	2	
20–29	23	5	
30+	58	73	
*Ethnicity*			0.30
Caucasian	55	52	
African Canadian	7	2	
Native/Aboriginal	1	3	
Arab	7	3	
Latin American	4	4	
Asian	6	8	
Other	5	8	
*Family situation*			0.71
Single-parent	11	13	
Double-parent	74	68	
Prefer not to answer	1	2	
*Yearly household income*			0.48
<20,000	10	9	
20–39,000	9	9	
40,000–59,000	7	7	
60,000–79,000	2	8	
80,000–99,000	6	8	
100,000–119,000	7	6	
120,000–149,000	6	5	
More than 150,000	7	12	
*Highest level of education*			0.71
Elementary school	3	1	
Secondary school	10	13	
College	21	26	
University	35	27	
Graduate school	13	13	

*Note.* Frequencies (*n*) unless otherwise noted. Groups were compared using independent *t*-tests for continuous variables and Pearson Chi-square tests for categorical variables.

**Table 2:** Means (standard deviations) of physical activity intensities and sedentary time (min/h) for participating children in the Childcare PLAY policy.

Time	LPA		MVPA		TPA		Sedentary Time	
	Control	Exp	Control	Exp	Control	Exp	Control	Exp
Baseline	10.04 (1.73)	9.59 (2.04)	9.38 (3.13)	8.74 (3.71)	19.42 (4.63)	18.33 (5.43)	40.58 (4.63)	41.67 (5.43)
Mid	9.35 (1.86)	9.68 (2.02)	8.35 (3.03)	8.40 (3.47)	17.70 (4.57)	18.08 (5.20)	42.30 (4.57)	41.92 (5.20)
Post	9.32 (1.84)	9.79 (2.03)	9.11 (3.21)	8.70 (3.72)	18.43 (4.64)	18.49 (5.25)	41.57 (4.64)	41.51 (5.25)
6-Month	9.92 (1.53)	10.66 (1.88)	10.08 (2.83)	9.86 (4.10)	20.00 (3.88)	20.52 (5.40)	40.00 (3.88)	39.48 (5.40)

*Note.* LPA: light physical activity; MVPA: moderate-to-vigorous physical activity; TPA: total physical activity; Exp. experimental condition. Reported differences for each time period represent the difference between groups.

## Data Availability

Not applicable.

## Supplementary Materials

The following are available online at https://www.mdpi.com/article/10.3390/ijerph18147468/s1, Figure S1: The Childcare PhysicaL ActivitY (PLAY) Policy.

## Abbreviations

BMI	Body mass index
ECE	Early childhood educator
CSEP	Canadian Society for Exercise Physiology
LPA	Light physical activity
MVPA	Moderate-to-vigorous physical activity
PLAY	Physical Activity
RCT	Randomized controlled trial
REB	Research ethics board
TPA	Total physical activity

## References

[1] Timmons B.W., Leblanc A.G., Carson V., Gorber S.C., Dillman C., Janssen I., Kho M.E., Spence J.C., Stearns J.A., Tremblay M.S. (2012). Systematic review of physical activity and health in the early years (aged 0–4 years). Appl. Physiol. Nutr. Metab..

[2] Carson V., Lee E.-Y., Hewitt L., Jennings C., Hunter S., Kuzik N., Stearns J.A., Unrau S.P., Poitras V.J., Gray C. (2017). Systematic review of the relationships between physical activity and health indicators in the early years (0–4 years). BMC Public Health.

[3] Colley R.C., Garriguet D., Adamo K.B., Carson V., Janssen I., Timmons B.W., Tremblay M.S. (2013). Physical activity and sedentary behavior during the early years in Canada: A cross-sectional study. Int. J. Behav. Nutr. Phys. Act..

[4] Leblanc A.G., Spence J.C., Carson V., Connor Gorber S., Dillman C., Janssen I., Kho M.E., Stearns J.A., Timmons B.W., Tremblay M.S. (2012). Systematic review of sedentary behaviour and health indicators in the early years (aged 0–4 years). Appl. Physiol. Nutr. Metab..

[5] Poitras V.J., Gray C.E., Janssen X., Aubert S., Carson V., Faulkner G., Goldfield G.S., Reilly J.J., Sampson M., Tremblay M.S. (2017). Systematic review of the relationships between sedentary behaviour and health indicators in the early years (0–4 years). BMC Public Health.

[6] Reilly J.J., Jackson D.M., Montgomery C., Kelly L.A., Slater C., Grant S., Paton J.Y. (2004). Total energy expenditure and physical activity in young Scottish children: Mixed longitudinal study. Lancet.

[7] Canadian Society for Exercise Physiology (2017). Canadian 24-Hour Movement Guidelines for the Early Years (0-4 years): An Integration of Physical Activity, Sedentary Behaviour and Sleep. BMC Public Health.

[8] Tremblay M.S., Chaput J.-P., Adamo K.B., Aubert S., Barnes J.D., Choquette L., Duggan M., Faulkner G., Goldfield G.S., Gray C.E. (2017). Canadian 24-Hour Movement Guidelines for the Early Years (0–4 years): An Integration of Physical Activity, Sedentary Behaviour, and Sleep. BMC Public Health.

[9] World Health Organization (2019). WHO Guidelines on Physical Activity, Sedentary Behaviour and Sleep for Children under 5 Years of Age.

[10] Copeland K.A., Khoury J.C., Kalkwarf H.J. (2016). Child care center characteristics associated with preschoolers’ physical activity. Am. J. Prev. Med..

[11] Vanderloo L.M., Tucker P., Johnson A.M., Van Zandvoort M.M., Burke S.M., Irwin J.D. (2014). The influence of centre-based childcare on preschoolers’ physical activity levels: A cross-sectional study. Int. J. Env. Res. Public Health.

[12] O’Brien K.T., Vanderloo L.M., Bruijns B.A., Truelove S., Tucker P. (2018). Physical activity and sedentary time among preschoolers in centre-based childcare: A systematic review. Int. J. Behav. Nutr. Phys. Act..

[13] Tucker P., Vanderloo L.M., Burke S.M., Irwin J.D., Johnson A.M. (2015). Prevalence and influences of preschoolers’ sedentary behaviors in early learning centers: A cross-sectional study. BMC Pediatr..

[14] Vanderloo L.M. (2014). Screen-viewing among preschoolers in childcare: A systematic review. BMC Public Health.

[15] Thompson D.A., Christakis D.A. (2005). The association between television viewing and irregular sleep schedules among children less than 3 years of age. Pediatrics.

[16] Simonato I., Janosz M., Archambault I., Pagani L.S. (2018). Prospective associations between toddler televiewing and subsequent lifestyle habits in adolescence. Prev. Med..

[17] Hesketh K.R., Lakshman R., Sluijs E.M.F. (2017). Barriers and facilitators to young children’s physical activity and sedentary behaviour: A systematic review and synthesis of qualitative literature. Obes. Rev..

[18] Robinson L.E., Webster E.K., Logan S.W., Lucas W.A., Barber L.T. (2011). Teaching practices that promote motor skills in early childhood settings. Early Child. Educ. J..

[19] Vanderloo L.M., Tucker P. (2018). Physical Activity and Sedentary Behavior Legislation in Canadian Childcare Facilities: An Update. BMC Public Health.

[20] Vercammen K.A., Frelier J.M., Poole M.K., Kenney E.L. (2020). Obesity prevention in early care and education: A comparison of licensing regulations across Canadian provinces and territories. J. Public Health.

[21] Ott E., Vanderloo L.M., Tucker P. (2019). Physical activity and screen-viewing policies in Canadian childcare centers. BMC Public Health.

[22] Bell A.C., Finch M., Wolfenden L., Fitzgerald M., Morgan P.J., Jones J., Freund M., Wiggers J. (2015). Child physical activity levels and associations with modifiable characteristics in centre-based childcare. Aust. N. Z. J. Public Health.

[23] Gerritsen S., Morton S.M.B., Wall C.R. (2016). Physical activity and screen use policy and practices in childcare: Results from a survey of early childhood education services in New Zealand. Aust. N. Z. J. Public Health.

[24] O’Neill J., Dowda M., Neelon S.E., Neelon B., Pate R. (2017). Effects of a new state policy on physical activity practices in child care centers in South Carolina. Am. J. Public Health.

[25] Bower J.K., Hales D.P., Tate D.F., Rubin D.A., Benjamin S.E., Ward D.S. (2008). The Childcare Environment and Children’s Physical Activity. Am. J. Prev. Med..

[26] Stephens R.L., Xu Y., Lesesne C.A., Dunn L., Kakietek J., Jernigan J. (2014). Relationship Between Child Care Centers’ Compliance with Physical Activity Regulations and Children’s Physical Activity, New York City, 2010. Prev. Chronic. Dis..

[27] Zhou Z., Ren H., Yin Z., Wang L., Zhixiong Z. (2014). A policy-driven multifaceted approach for early childhood physical fitness promotion: Impacts on body composition and physical fitness in young Chinese children. BMC Pediatr..

[28] Dowda M., Brown W.H., McIver K.L., Pfeiffer K.A., O’Neill J.R., Addy C.L., Pate R.R. (2009). Policies and Characteristics of the Preschool Environment and Physical Activity of Young Children. Pediatrics.

[29] Tandon P.S., Saelens B.E., Zhou C. (2018). A Comparison of Preschoolers’ Physical Activity Indoors versus Outdoors at Child Care. Int. J. Environ. Res. Public Health.

[30] Stacey F.G., Finch M., Wolfenden L., Grady A., Jessop K., Wedesweiler T., Bartlem K., Jones J., Sutherland R., Vandevijvere S. (2017). Evidence of the Potential Effectiveness of Centre-Based Childcare Policies and Practices on Child Diet and Physical Activity: Consolidating Evidence from Systematic Reviews of Intervention Trials and Observational Studies. Curr. Nutr. Rep..

[31] Staiano A.E., Webster E.K., Allen A.T., Jarrell A.R., Martin C.K. (2018). Screen-Time Policies and Practices in Early Care and Education Centers in Relationship to Child Physical Activity. Child. Obes..

[32] Government of Ontario (2014). Child Care and Early Years Act. https://www.ontario.ca/laws/statute/14c11.

[33] Tucker P., Driediger M., Vanderloo L.M., Burke S.M., Irwin J.D., Johnson A.M., Shelley J., Timmons B.W. (2019). Exploring the Feasibility and Effectiveness of a Childcare PhysicaL ActivitY (PLAY) Policy: Rationale and Protocol for a Pilot, Cluster-Randomized Controlled Trial. Int. J. Environ. Res. Public Health.

[34] McClain J.J., Tudor-Locke C. (2009). Objective monitoring of physical activity in children: Considerations for instrument selection. J. Sci. Med. Sport..

[35] Engelen L., Bundy A.C., Naughton G., Simpson J.M., Bauman A., Ragen J., Baur L., Wyver S., Tranter P., Niehues A. (2013). Increasing physical activity in young primary school children—It’s child’s play: A cluster randomised controlled trial. Prev. Med..

[36] Alhassan S., Laurent CSt Burkart S., Greever C.J., Ahmadi M. (2018). Effects of integrating physical activity into early education learning standards on preschoolers’ physical activity levels. Med. Sci. Sport Exerc..

[37] Pate R.R., Almeida M.J., McIver K.L., Pfeiffer K.A., Dowda M. (2006). Validation and calibration of an accelerometer in preschool children. Obesity.

[38] Kuczmarski R.J., Ogden C.L., Guo S.S., Grummer-Strawn L.M., Flegal K.M., Mei Z., Wei R., Curtin L.R., Roche A.F., Johnson C.L. (2002). 2000 CDC Growth Charts for the United States: Methods and development. Vital Health Stat.

[39] Witjzes A.L., Kooijman M.N., Kiefte-de Jong J.C., de Vries S.I., Henrichs J., Jansen W., Jaddoe V.W., Hofman A., A Moll H., Raat H. (2013). Correlates of physical activity in 2-year-old toddlers: The Generation R Study. J. Pediatr..

[40] Bunn A., Korpela M. (2019). R Core Team. R: A Language and Environment for Statistical Computing.

[41] Pinheiro J., Bates D., DebRoy S., Sarkar D. (2021). Nlme: Linear and Nonlinear Mixed Effects Models. https://CRAN.R-project.org/package=nlme.

[42] Fox J., Weisberg S. (2019). An {R} Companion to Applied Regression.

[43] Lenth R. (2020). Emmeans: Estimaed Marginal Means, Aka Least-Squares, Version 1.4. 4. https://cran.r-project.org/web/packages/emmeans/emmeans.pdf.

[44] Wolfenden L., Jones J., Williams C.M., Finch M., Wyse R.L., Kingsland M., Tzelepsis F., Wiggers J., Williams A.J., Seward K. (2015). Strategies to improve the implementation of healthy eating, physical activity and obesity prevention policies, practices or programmes within childcare services. Cochrane Database Syst. Rev..

[45] Sterdt E., Pape N., Kramer S., Urban M., Werning R., Walter R. (2013). Do preschools differ in promoting children’s physical activity? An instrument for the assessment of preschool physical activity programmes. BMC Public Health.

[46] Wolfenden L., Neve M., Farrell L., Lecathelinais C., Bell C., Milat A., Wiggers J., Sutherland R. (2011). Physical activity policies and practices of childcare centres in Australia. J. Paediatr. Child Health.

[47] Breck A., Goodman K., Dunn L., Stephens R.L., Dawkins N., Dixon B., Jernigan J., Kakietek J., Lesesne C., Lessard L. (2014). Evaluation design of New York City’s regulations on nutrition, physical activity, and screen time in early child care centers. Prev. Chronic. Dis..

[48] LaRowe T.L., Tomayko E.J., Meinen A.M., Hoiting J., Saxler C., Cullen B. (2016). Active Early: One-year policy intervention to increase physical activity among early care and education programs in Wisconsin. BMC Public Health.

[49] Henderson K.E., Grode G.M., O’Connell M.L., Schwartz M.B. (2015). Environmental factors associated with physical activity in childcare centers. Int. J. Behav. Nutr. Phys. Act..

[50] Vanderloo L.M., Tucker P., Johnson A.M., Holmes J.D. (2013). Physical activity among preschoolers during indoor and outdoor childcare play periods. Appl. Physiol. Nutr. Metab..

[51] Alhassan S., Nwaokelemeh O., Mendoza A., Shitole S., Puleo E., Pfeiffer K.A., Whitt-Glover M.C. (2016). Feasibility and Effects of Short Activity Breaks for Increasing Preschool-Age Children’s Physical Activity Levels. J. Sch. Health.

[52] Driediger M., Truelove S., Johnson A.M., Vanderloo L.M., Timmons B.W., Burke S.M., Irwin J.D., Tucker P. (2019). The impact of shorter, more frequent outdoor play periods on preschoolers’ physical activity during childcare: A cluster randomized controlled trial. Int. J. Env. Res. Public Health.

[53] Wolfenden L., Wiggers J., Morgan P., Razak L.A., Jones J., Finch M., Sutherland R., Lecathenlinais C., Gillham K., Yoong S.L. (2016). A randomised controlled trial of multiple periods of outdoor free-play to increase moderate-to-vigorous physical activity among 3 to 6 year old children attending childcare: Study protocol. BMC Public Health.

[54] Tucker P., Vanderloo L.M., Johnson A.M., Burke S.M., Irwin J.D., Gaston A., Driediger M., Timmons B.W. (2017). Impact of the Supporting Physical Activity in the Childcare Environment (SPACE) intervention on preschoolers’ physical activity levels and sedentary time: A single-blind cluster randomized controlled trial. Int. J. Behav. Nutr. Phys. Act..

[55] Szpunar M., Johnson A.M., Driediger M.V., Burke S.M., Irwin J.D., Shelley J., Timmons B.W., Vanderloo L.M., Tucker P. (2021). Implementation adherence and perspectives of the childcare PhysicaL ActivitY (PLAY) policy: A process evaluation. Health Educ Behav..

[56] Gubbels J.S., Kremers S.P., van Kann D.H., Stafleu A., Candel M.J., Dagnelie P.C. (2011). Interaction between physical environment, social environment, and child characteristics in determining physical activity at childcare. Health Psychol..

