# Supplementary material for: Impact of the Childcare Physical Activity (PLAY) Policy on Young Children’s Physical Activity and Sedentary Time: A Pilot Clustered Randomized Controlled Trial
Source: Int J Environ Res Public Health. 2021 Jul 13;18(14):7468. doi: 10.3390/ijerph18147468 (PMC8304787; doi:10.3390/ijerph18147468)
Supplement: Supplementary file 1 [file ijerph-18-07468-s001.zip › ijerph-1289590-supplementary.pdf]

Figure S1: The Childcare PhysicaL ActivitY (PLAY) Policy

The PhysicaL ActivitY (PLAY) Policy

Directed by the Canadian 24-Hour Movement Guidelines for the Early Years*, childcare programs are expected to:

1. Encourage children to engage in higher intensity energetic play (i.e., activities that induce sweating and heavy breathing) often throughout the day with a goal of accumulating a *minimum of 40 minutes each day*. More is better.
2. Expose children to a variety of *indoor* and *outdoor* physical activities, including both child-directed and teacher-facilitated active play daily.
3. Outdoor time is offered for a *minimum of 120 minutes each day* unless *extreme* weather (i.e., heat or cold alert) prevents it. When extreme weather occurs, the opportunity exists for children to engage in active play indoors.
4. Short, frequent outdoor sessions are most conducive to higher intensity physical activity among children; therefore, short bouts (e.g., 15-30 minutes) of outdoor time are recommended often (e.g., 3-4 times a day).
5. Unstructured (i.e., child-directed) free play is predominant during outdoor time. When activity levels decline, childcare practitioners encourage continued energetic play through structured activity, participation alongside children, and use of verbal prompts.
6. Encourage children to develop physical literacy by practicing fundamental movement skills often throughout the day (e.g., running, skipping, hopping, or jumping).
7. The appropriate use of screen-based technology is role modelled by childcare providers by avoiding it when children are present. Screen-based technology is not offered to children under 2, and is not recommended during childcare hours.
8. Programming is designed to break up sustained sedentary time using indoor movement based activities.

.

Supplementary File: Informed Consent Form

PLAY (PhysicaL ActivitY) Policy Study
Examining a Childcare Policy to Promote Physical Activity among Young Children

I have read the Letter of Information, have had the nature of the study explained to me, and I agree to participate. All questions have been answered to my satisfaction.

________	______________________________	___________________________	________________________
Date	Participant's Name<br>(please print)	Parent/Guardian Name<br>(please print)	Parent/Guardian Signature

________	______________________________	___________________________
Date	Name of Researcher Obtaining Informed Consent<br>(please print)	Signature

Do you wish to obtain a copy of the study results?

Yes
No

If YES, please provide your email address below

Email: ________________________________
